# A universal strategy for high-yield production of soluble and functional clostridial collagenases in *E. coli*

**DOI:** 10.1007/s00253-009-1953-4

**Published:** 2009-03-31

**Authors:** Paulina Ducka, Ulrich Eckhard, Esther Schönauer, Stefan Kofler, Gerhard Gottschalk, Hans Brandstetter, Dorota Nüss

**Affiliations:** 1 Department of Molecular Biology, Division of Structural Biology, University of Salzburg, Billrothstraße 11, 5020 Salzburg, Austria; 2 Institute of Microbiology and Genetics, Georg-August-University Göttingen, Grisebachstraße 8, 37077 Göttingen, Germany

**Keywords:** Clostridial collagenases, Expression, Purification, Platform

## Abstract

Clostridial collagenases are foe and friend: on the one hand, these enzymes enable host infiltration and colonization by pathogenic clostridia, and on the other hand, they are valuable biotechnological tools due to their capacity to degrade various types of collagen and gelatine. However, the demand for high-grade preparations exceeds supply due to their pathogenic origin and the intricate purification of homogeneous isoforms. We present the establishment of an *Escherichia coli* expression system for a variety of constructs of collagenase G (ColG) and H (ColH) from *Clostridium histolyticum* and collagenase T (ColT) from *Clostridium tetani*, mimicking the isoforms in vivo. Based on a setup of five different expression strains and two expression vectors, 12 different constructs were expressed, and a flexible purification platform was established, consisting of various orthogonal chromatography steps adaptable to the individual needs of the respective variant. This fast, cost-effective, and easy-to-establish platform enabled us to obtain at least 10 mg of highly pure mono-isoformic protein per liter of culture, ideally suited for numerous sophisticated downstream applications. This production and purification platform paves the way for systematic screenings of recombinant collagenases to enlighten the biochemical function and to identify key residues and motifs in collagenolysis.

## Introduction

Clostridia comprise a diverse family of anaerobic, sporulating bacteria, including notorious pathogenic species such as *Clostridium botulinum*, *Clostridium perfringens*, and *Clostridium difficile*. Two further prominent representatives are *Clostridium histolyticum*, a pathogen-causing gas gangrene, and *Clostridium tetani* giving rise to tetanus (Bruggemann et al. 2003; Burke and Opeskin 1999; Sasaki et al. 2000). While the histotoxicity of Clostridia is primarily caused by specific toxins (Hatheway 1990), host infiltration and colonization are triggered by the production of various proteases such as collagen degrading zinc-metalloproteases, namely collagenases (Bruggemann and Gottschalk 2004; Hatheway 1990; Mallya et al. 1992) and polysaccharide— and lipid—degrading enzymes (Matsushita and Okabe 2001). Therefore, clostridial collagenases have been proposed as important drug-target candidates.

Additionally, with collagens being the most abundant proteins in all higher organisms, there exists a diverse spectrum of therapeutic and biotechnological applications for bacterial collagenases, in particular for the biochemically well-characterized ColG and H from *C. histolyticum*, including their use for islet cell isolation, wound healing, treatment of retained placenta, or their use as additives to laundry detergents, to name a few (Chu 1987; Haffner et al. 1998; Hesse et al. 1995; Sank et al. 1989; Watanabe 2004).

**Fig. 1:**
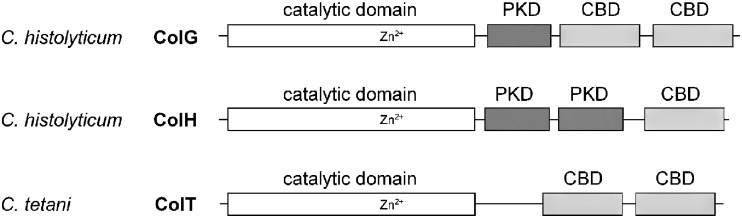
Schematic representation of the domain architecture of three different mature clostridial collagenases. *PKD* polycystic kidney disease domain; *CBD* collagen binding domain

All currently classified clostridial collagenases are members of the MEROPS peptidase subfamily M9B (Rawlings et al. 2006). They are mosaic proteins consisting of a signal peptide, a catalytic domain, up to two polycystic kidney disease domain(s) (PKD) of unknown function and up to three collagen binding domain(s) (CBD; see Fig. 1). Their domain organization varies significantly. For example, ColG and ColA from *C. perfringens* possess a duplicated CBD and a single PKD. In ColH, the latter is duplicated, whereas it is completely absent in ColT from *C. tetani* (Bruggemann and Gottschalk 2004; Watanabe 2004).

Contrasting the zinc coordination in metzincins (e.g., mammalian collagenases), bacterial collagenases as gluzincins employ a glutamate as third catalytic zinc binding ligand next to the two histidines in the canonical HEXXH motif of the zincin superfamily (Jung et al. 1999). The structural knowledge about the clostridial collagenases is poor and currently confined to the crystal structure of the CBD (Wilson et al. 2003) that has been shown to bind to collagen in a cooperative and calcium dependent manner (Matsushita et al. 1998; Toyoshima et al. 2001; Wilson et al. 2003).

The high biotechnological and medical interest in clostridial collagenases that is mirrored by an increasing demand for high grade mono-isoformic preparations, in particular of ColG and ColH, faces a shortage of supply due to (1) the pathogenic origin of ColH and ColG and (2) the intricacy associated with the purification of homogeneous isoforms. The development of simple and efficient high-level expression and purification strategies would also facilitate basic and applied research of bacterial collagenases. Reported recombinant expression trials focused on *Bacillus subtilis*, *C. perfringens*, and *Escherichia coli* as expression systems. (1) *Bacillus subtilis* enabled the secretory expression of ColG and ColH, but the expression system suffered from plasmid instability and low protein yields (Jung et al. 1996). The latter was attributed to the remarkably high A + T content of clostridial genes and their biased codon usage (Sharp et al. 2005; Tanaka et al. 2008). (2) Bypassing these drawbacks associated with heterologous expression, Tanaka et al. developed a protease-deficient *C. perfringens* strain 13 expression system and were able to purify mg amounts of homogeneous ColH (Tamai et al. 2008; Tanaka et al. 2008). (3) Already in 1995, Hesse et al. utilized non-pathogenic *E. coli* strains for the recombinant expression of clostridial collagenases, exploiting the short generation times, easy handling and the well established fermentation know-how of this host, resulting in expression yields several fold higher than in the natural host (Hesse et al. 1995).

Although the latter expression system was considered as inefficient in translating clostridial genes (Tanaka et al. 2008), we report the production and purification of mg amounts of high-grade mono-isoformic clostridial collagenases preparations on a small laboratory scale. The established protein expression and purification platform provides cost-efficient access to these biotechnologically important enzymes and paves the way for systematic enzyme engineering approaches.

**Fig. 2:**
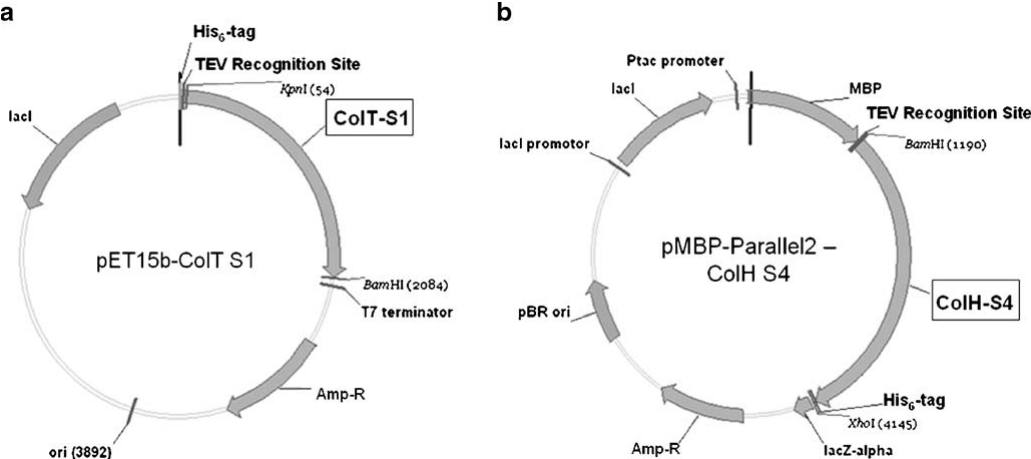
Schematic representation of the expression vectors. **a** pET15b and **b** pMBP-Parallel2

In our study, we selected three bacterial collagenases: ColG, H, and T that are characterized by complementary domain architectures (Fig. 1). A variety of constructs (Table 1), reflecting this mosaicity, was designed and combined with two types of expression plasmid (Fig. 2): in the first, the recombinant proteins were fused to an N-terminal His_6_-tag; the second plasmid allowed a tandem-tag strategy, combining an N-terminal cleavable maltose binding protein (MBP)-tag and a C-terminal His_6_-tag. These tagging variants facilitated highly efficient purification strategies that allowed us to achieve both high yield (at least 10 mg/l of culture range) and homogeneous (>95% based on Coomassie stained SDS-PAGE) protein preparations.

**Table 1:** Constructs of ColG, H and T and expression parameters

Vector	Construct		Fusiontag(s)		Start	End	MW	*E. coli*	POI	[IPTG]	Expr.	POE
			MBP	His_6_	sequence	sequence	(kDa)	strain	(OD_600_)	(mM)	Temp. (°C)	
pET15b	Co1G	hG4: S1S2S3a3b		N-TEV	Y^119^ DF	VNK^1118^	115.6	BL21 DE3	0.8	0.1	37	4 h
		hG3: S1S2S3a				NIK^1001^	102.2		0.8	0.1	37	4 h
		hG2: S1S2				IKN^880^	88.9		1.2	0.1	25	4 h
		hG1: S1				DNG^790^	79.4		1.2	0.1	25	4 h
	Co1H	hH4: S1S2aS2bS3		N-TEV	V^41^ QN	NIE^1016^	114.1	BL21 DE3	1.2	0.1	37	ON
		hH3: S1S2aS2b				ITD^900^	101.2		1.2	0.1	25	ON
		hH2: S1S2a				IRD^810^	91.3		1.2	0.1	25	ON
		hH1: S1				GYL^717^	81.0		0.8	0.1	25	4 h
		hH0“mini”				GYL^556^	59.8		0.8	0.1	37	4 h
	Co1T	hT3: S1S3aS3b		N-TEV	Y^53^ KT	IIN^991^	111.3	Tuner DE3	1.2	0.1	37	ON
		hT1: S1				GLL^727^	80.9		0.8	0.1	25	4 h
		hT0“mini”				FFA^506^	53.0		0.8	0.1	25	4 h
pMBP-Parallel2	Co1G	mG4: S1S2S3a3b	N-TEV	C	Y^119^ DF	VNK^1118^	158.0	BL21	1.2	1.0	25	ON
		mG3: S1S2S3a				NIK^1001^	144.7		1.2	1.0	25	ON
		mG2: S1S2				IKN^880^	131.3		1.2	1.0	25	ON
	Co1H	mH4: S1S2aS2bS3	N-TEV	C	V^41^ QN	NIE^1016^	156.0	BL21	1.2	1.0	25	ON
		mH3: S1S2aS2b				ITD^900^	143.0		1.2	1.0	25	ON
		mH2: S1S2a				IRD^810^	133.0		1.2	1.0	25	ON
	Co1T	mT3: S1S3a3b	N-TEV	C	Y^53^ KT	IIN^991^	153.0	BL21	1.2	1.0	25	ON

Lowercase letter indicates the cleavable N-terminal tag (h: His6, m: MBP), capital letter the respective collagenase (G, H or T), and the digit denotes the number of domains present in the construct. *S1* catalytic domain, *S2* PKD; *S3* CBD; *N–TEV* N-terminal tag followed by a TEV cleavage site; *C* non-cleavable C-terminal tag; *MW* molecular weight; *POI* point of induction; [*IPTG*], final IPTG concentration; *Expr. Temp* expression temperature; *POE* period of expression

## Materials and methods

### Materials

Plasmids encoding for ColG (GenBank: D87215.1) and ColH (GenBank: D29981.1) were obtained from Roche Diagnostics, Penzberg (Germany). The genomic DNA of *Clostridium tetani* strain E88 (GenBank: NC_004557) was obtained from Göttingen Genomics Laboratory, Göttingen (Germany).

Restriction enzymes and T4 Ligase were obtained from Fermentas, St. Leon-Rot (Germany). Pfu Ultra II™ Fusion HS DNA Polymerase was obtained from Stratagene, La Jolla (USA). Custom-made primers were obtained from Eurofins MWG Operon, Ebersberg (Germany), and sequence analyses were performed at Eurofins MWG Operon, Martinsried (Germany).

*E. coli* strain XL1 Blue (Stratagene, La Jolla, USA) was used for subcloning. Strains BL21, BL21 DE3, Tuner DE3, Origami DE3, and Rosetta DE3 (Novagen, Madison, USA) were used as host strains for protein expression. For expression, LB-Lennox (Roth, Karlsruhe, Germany) was used. All reagents were of the highest standard available from Sigma–Aldrich Co., München (Germany) or AppliChem, Darmstadt (Germany).

### Cloning

The encoding DNA fragments were amplified by polymerase chain reaction (PCR; Eppendorf mastercycler ep gradient thermal cycler) using genomic DNA (ColT) or plasmid DNA (ColG and H) as template and appropriate primer containing the restriction sites for subsequent cloning (Tables 2 and 3). PCR products were purified with MinElute PCR Purification Kit (Qiagen, Hilden, Germany) and digested with the appropriate restriction enzymes, ligated under standard conditions, and introduced into XL1 Blue cells via electroporation by standard protocols. All inserts were cloned in a modified pET15b expression vector encoding for an N-terminal His_6_-tag followed by a TEV (Tobacco Etch Virus protease) cleavage site for specific tag removal. Constructs intended for tandem affinity purification via a TEV-cleavable N-terminal MBP-tag and a non-cleavable C-terminal His_6_-tag were cloned in the expression vector pMBP-Parallel2 (Sheffield et al. 1999). All constructs were confirmed by DNA sequencing prior to protein expression.

### Expression of ColG, H, and T

#### Test expression and solubility testing

Plasmids were introduced into expression hosts via electroporation. Three milliliter of LB media containing the appropriate antibiotics were inoculated with a single bacterial colony from a fresh LB-agar plate and incubated at 37 °C with vigorous shaking (230 rpm) overnight. 50 µl of the overnight cultures were diluted 1:1,000 in fresh LB medium, containing the appropriate antibiotics, and incubated at 37 °C with shaking in 250-ml baffled flasks until the bacterial cultures reached the planned optical density. After induction, cultures were transferred to the respective temperature and allowed to grow for at least additional 4 h. Cells were harvested by centrifugation for 20 min at 5,000 × *g* and 4 °C. Parameters considered for optimization in terms of maximizing soluble expression were: (1) different *E. coli* strains (given above), (2) final isopropyl-ß-d-thiogalactoside (IPTG) concentrations (0.1, 1.0 mM), (3) cell densities at point of induction (OD_600_ 0.8, 1.2), (4) expression temperatures (25 and 37 °C), and (4) duration of expression (4 h, ON). In sum, 64 different expression conditions were tested for every construct. Parameters found optimal were used for large-scale expressions.

#### Large-scale expression and cell harvest

Large-scale expression was carried out in the respective *E. coli* strains under the identified conditions (Table 1). Cells were harvested by centrifugation for 20 min at 4,000×*g* and 4 °C. Pellets were resuspended in a buffer containing 50 mM NaH_2_PO_4_, 10 mM Tris, 150 mM NaCl, 10 mM imidazole, pH 8.0, and subsequently sonicated intermittently on ice (5 × 30 s, 45 W). Cell debris was removed by centrifugation (×2) for 30 min at 15,000 × *g* and 4 °C.

### Purification and activity assay of ColG, ColH, and ColT

All purification steps were performed at 4 °C or with precooled buffers.

#### Immobilized metal affinity chromatography

Purification was carried out in batch mode with pre-equilibrated (50 mM NaH_2_PO_4_, 300 mM NaCl, 10 mM imidazole, pH 8.0) Ni-NTA Superflow resin (Qiagen, Hilden, Germany). Cleared lysate was loaded onto the resin and washed at least twice with buffer containing various imidazole concentrations (buffer given above with 20–40 mM imidazole). Target protein was eluted in a single step with a high imidazole buffer (250 mM imidazole).

#### Amylose affinity chromatography

Purification via the MBP tag was carried out in batch mode. Amylose resin (New England BioLabs, Frankfurt, Germany) was pre-equilibrated with MBP-purification buffer (20 mM Tris, 200 mM NaCl, pH 7.5), protein was loaded and washed at least twice with MBP purification buffer. MBP-tagged protein was eluted with the buffer given above supplemented with 10 mM maltose.

#### Removal of the N-terminal tag

The N-terminal tag was removed using the Tobacco Etch Virus protease in a molar ratio of 1:5 or 1:20 (enzyme to target protein) in a buffer containing 50 mM Tris, 50 mM NaCl, 1 mM EDTA, and 2 mM DTT at pH 7.5 and 4 °C for 12 to 48 h. To check the completeness of TEV digest, a re-chromatography on a Ni-NTA column in batch format was performed.

#### Ion exchange chromatography

For ion exchange chromatography (IEC), the ÄKTA FPLC system and a Q-Sepharose Column (HiPrep™ 16/10 Q FF; GE Healthcare) were used. The protein sample was rebuffered via dialysis into salt-free buffer and filtered before loading onto the pre-equilibrated column (50 mM MES, pH 6.5). Application of the sample occurred at a flow rate of 0.2 ml/min to alleviate binding. The target protein was eluted with a high salt and low pH buffer (50 mM MES, 1.0 M CaCl_2_, pH 4.0) using a step gradient at a flow rate of 0.6 ml/min.

#### Size exclusion chromatography

As final polishing step, the concentrated (by ultrafiltration) and filtrated protein sample was loaded onto a Superdex 200 10/300 GL (GE Healthcare) column on the ÄKTA FPLC system. The buffer used for size exclusion chromatography (SEC) contained 25 mM Tris, 50 mM NaCl, pH 7.5.

#### Activity assay

The biological activity of the clostridial collagenases was tested after Ni-NTA purification using *N*-(3-[2-Furyl]-Acryloyl)-Leu-Gly-Pro-Ala (FALGPA) as a synthetic substrate (Van Wart and Steinbrink 1981).

### SDS-PAGE and protein quantitation

Routinely, expression and purification results were monitored by SDS-PAGE. Samples were resuspended in or mixed with 2×SDS buffer and separated by SDS-PAGE. Depending on the molecular weight of the target protein, 10%, 12%, or 15% (*w*/*v*) polyacrylamide gels were used. A prestained protein ladder was used as size marker (Fermentas), and gels were stained with Coomassie Brilliant Blue R-250. Protein concentrations were determined by the Bradford dye binding assay with bovine serum albumin as standard (Bradford 1976) and/or UV_280_ measurements.

## Results

### Construct design and cloning

To gain insights into the structure–function relationship of the clostridial collagenases G, H, and T, the proteins were dissected by constructing different recombinant derivatives of the full length enzymes. To delineate the appropriate boundaries of the different domains, we combined the following data: (1) information on the mature N-terminus as described in the literature (Matsushita and Okabe 2001), (2) information on naturally occurring isoforms, as described for ColG and ColH from *C. histolyticum* (Bond and Van Wart 1984a, b; Matsushita et al. 1999), and ColA from *C. perfringens* (Matsushita et al. 1994), and (3) bioinformatical analyses that included multiple sequence alignments and secondary structure predictions (Tables 2, 3). Thereby, the initial construct design mimics the domain organization of collagenase isoforms in vivo. A posteriori, two additional constructs were cloned in response to the presence of a truncated fragment approximately 25 kDa smaller than the catalytic domain construct in preparations of ColT: (1) a truncated variant of the catalytic domain of ColT (Tyr^53^–Ala^506^; 55 kDa), ending shortly after the third zinc binding glutamate and (2) based on the domain boarders predicted by the CHOP server (Liu and Rost 2004), a shortened variant of the catalytic domain of ColH (Val^41^–Leu^556^; 60 kDa).

**Table 2:** Sequences of oligonucleotide primers used for the cloning into pET15b vector

Vector	Construct		Primer	Sequence
pET15b	ColG	hG	Forward	KpnI site: 5′-ACGT GGTACC ATGTATGATTTTGAGTATTTAAATG-3′
		hG1: S1	Reversed	BamHI site: 5′-ACGT GGATCC TTACCCATTATCTGTTAAAACCC-3′
		hG2: S1S2	Reversed	BamHI site: 5′-ACGT GGATCC TTAGTTCTTTATTTCTATAGTAA-3′
		hG3: S1S2S3a	Reversed	BamHI site: 5′-ACGT GGATCC TTATCCTTTTATGTTTAAAGAAT-3′
		hG4: S1S2S3a3b	Reversed	BamHI site: 5′-ACGT GGATCC TTATTTATTTACCCTTAACTCAT-3′
	ColH	hH	Forward	KpnI site: 5′-ACGT GGTACC ATGGTACAAAATGAAAGTAAGAG-3′
		hH0 “mini”	Reversed	BamHI site: 5′-AGCA GGATCC CTATAATATACCCATATCTTTATTATAC-3′
		hH1: S1	Reversed	BamHI site: 5′-ACGT GGATCC TTATAAATATCCGTGGAATACTA-3′
		hH2: S1S2a	Reversed	BamHI site: 5′-ACGT GGATCC TTAATCTCTTATTTCTGCAGTAG-3′
		hH3: S1S2a2b	Reversed	BamHI site: 5′-ACGT GGATCC TTAATCTGTAATCTTAATCTTCA-3′
		hH4: S1S2a2bS3	Reversed	BamHI site: 5′-CCCC GGATCC TTATTCTATATTAATTCTATATG-3′
	ColT	hT	Forward	KpnI site: 5′-GG GGTACC TACAAAACAAAGTATTCTTTTAATG-3′
		hT0 “mini”	Reversed	XhoI site 5′-CCG CTCGAG TCATGCAAAAAATTCTGCAGAGCCTTC-3′
		hT1: S1	Reversed	BamHI site: 5′-CCG GGATCC TCATAATAATCCATGGAAAACTATATCATA -3′
		hT3: S1S3a3b	Reversed	XhoI site 5′-CCG CTCGAG TTAATTTATTATTACTGAATAATTACC-3′

Lowercase letter indicates the cleavable N-terminal tag (h: His_6_), capital letter the respective collagenase (G, H or T), and the digit gives/denotes the number of domains present in the construct. *S1* catalytic domain; *S2* PKD domain; *S3* CBD

**Table 3:**
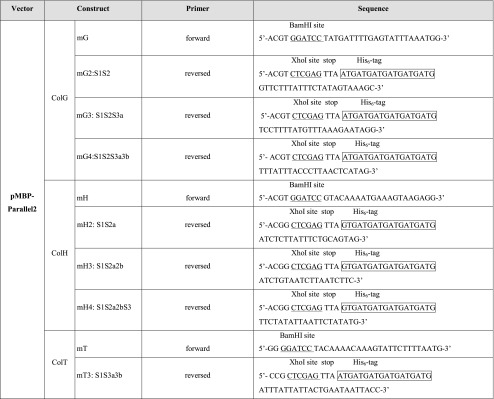
Sequences of oligonucleotide primers used for cloning into the pMBP-Parallel2 vector

Lowercase letter indicates the cleavable N-terminal tag (m: MBP), capital letter the respective collagenase (G, H or T), and the digit gives/denotes the number of domains present in the construct. *S1* catalytic domain; *S2* PKD domain; *S3* CBD

All constructs, four of ColG, five of H, and three of ColT (displayed in Table 1), were successfully cloned in a modified pET15b *E. coli* expression vector, encoding for an N-terminal His_6_-tag followed by a TEV recognition motif (Fig. 2a). Due to observed protein degradation during protein purification (data not shown) seven constructs, three of ColG, and ColH each and one of ColT were subcloned into the pMBP-Parallel2 vector (Sheffield et al. 2009), shown in Fig. 2b, encoding an N-terminal TEV-cleavable MBP-tag and a non-cleavable C-terminal His_6_-tag, allowing a tandem purification approach that results in clearly delimited protein samples.

### Optimization of ColG, H, and T protein expression in *E. coli*

**Fig. 3:**
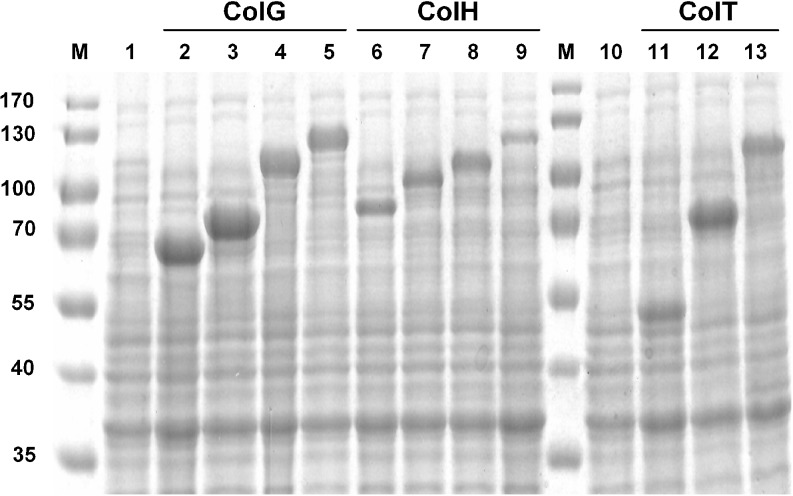
Expression of ColG, H, and T constructs cloned in pET15b. For the nomenclature, compare Table 1. *M* prestained protein ladder. *Lane 1* BL21 DE3 cells before induction; *lanes 2*–*5* hG1, hG2, hG3, and hG4 constructs of ColG 4 h after induction; *lanes 6*–*9* hH1, hH2, hH3, and hH4 constructs of ColH 4 h after induction; *lane 10* Tuner DE3 cells before induction; *lanes 11*–*13* hT0, hT1, and hT3 constructs of ColT

To maximize soluble protein expression, 64 different expression conditions, based on five different parameters (host strain, point of induction, final IPTG concentration, period, and temperature of expression) were screened for every construct. Detailed results of the optimized expression parameters are shown in Table 1. A representative expression gel of all constructs cloned in pET-15b is shown in Fig. 3.

To sum up, (1) all pET15b constructs of ColG and ColH were expressed in BL21 DE3, whereas ColT constructs showed highest soluble expression levels in Tuner DE3 cells. (2) For maximum soluble expression of the longer collagenase constructs, time of induction was switched from OD_600_ 0.8 to 1.2 and expression temperature was lowered to 25 °C to minimize the accumulation of protein in inclusion bodies. (3) Cells were induced with 0.1 mM IPTG in case of pET15b constructs for two simple reasons: lower IPTG concentrations significantly enhanced the output of soluble ColT protein, and it was economically favored for ColG and ColH constructs, because no difference in soluble expression yield could be observed upon increasing IPTG concentrations. (4) All pMBP-Parallel2 constructs were expressed in BL21 at 25 °C for a prolonged period and at a higher final concentration of IPTG. (5) In general, ColG showed highest expression levels, followed by ColT and ColH.

### Protein purification

**Fig. 4:**
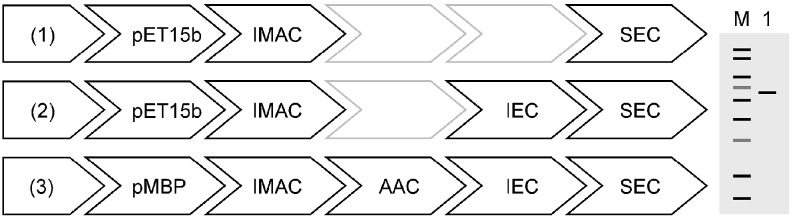
Schematic representation of the modular purification platform. (*1*) A simple two-step approach consisting of IMAC and SEC was employed for shorter constructs (hG1, hH0, hH1, hT0). (*2*) Constructs of intermediate length were purified in three steps using IMAC, IEC, and SEC (hG2, hG3, hH2, hH3, hT1). (*3*) Long and full-length constructs were cloned in the pMBP-Parallel2 vector and the initial IMAC step was followed by amylose affinity chromatography (*AAC*), prior to *IEC* and the final *SEC* step (mG3, mG4, mH3, mH4, mT3). For the nomenclature, compare also Table 1. *IMAC*, immobilized metal affinity chromatography; *AAC*, amylose affinity chromatography; *IEC*, ion exchange chromatography; *SEC*, size exclusion chromatography; *pMBP*, pMBP-Parallel2

To achieve pure and homogeneous protein samples, it was necessary to combine diverse purification methods (Fig. 4). We, therefore, established a flexible purification platform adaptable to the individual needs of the investigated constructs. We utilized not only the advantages of well-characterized fusion tags (His_6_- and MBP-tag) but also capitalized on intrinsic properties of the proteins, e.g., calcium binding properties and pI (approximately 5.5 for all constructs), for successful purification via IEC. As a final polishing step SEC was routinely used.

**Fig. 5:**
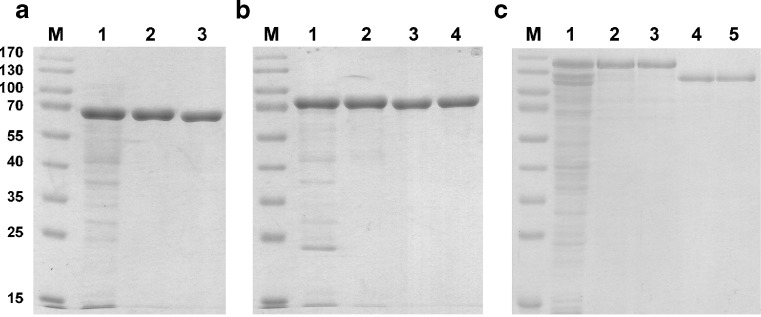
Differential purification strategy exemplified by hG1, hG2, and mG4. For the nomenclature, compare Table 1. *M* prestained protein ladder. **a** Purification of hG1. *Lane 1* cleared lysate; *lane 2* hG1 after NiNTA purification; *lane 3* hG1 without His_6_-tag after SEC. **b** Purification of hG2. *Lane 1* cleared lysate; *lane 2* hG2 after NiNTA purification; *lane 3* hG1 without His_6_-tag after IEC; *lane 4* hG1 without His_6_-tag after SEC. **c** Purification of mG4. *Lane 1* cleared lysate; *lane 2* mG4 after NiNTA purification; *lane 3* mG4 after purification via amylose resin. *Lane 4* mG4 without MBP-tag after IEC; *lane 5* mG4 without MBP-tag after SEC

For the catalytic domains of ColG and ColH a simple two-step approach consisting of a NiNTA and a SEC purification step was already sufficient to obtain pure protein (Fig. 5a). But in most cases, at least one additional orthogonal purification step via IEC had to be applied (Fig. 5b). For the full length constructs, even a tandem affinity approach combined with IEC and SEC was necessary to successfully bypass co-purification of C-terminally degraded variants (Fig. 5c). N-terminal fusion tags were routinely removed with TEV protease before or after the ion exchange chromatography step.

**Fig. 6:**
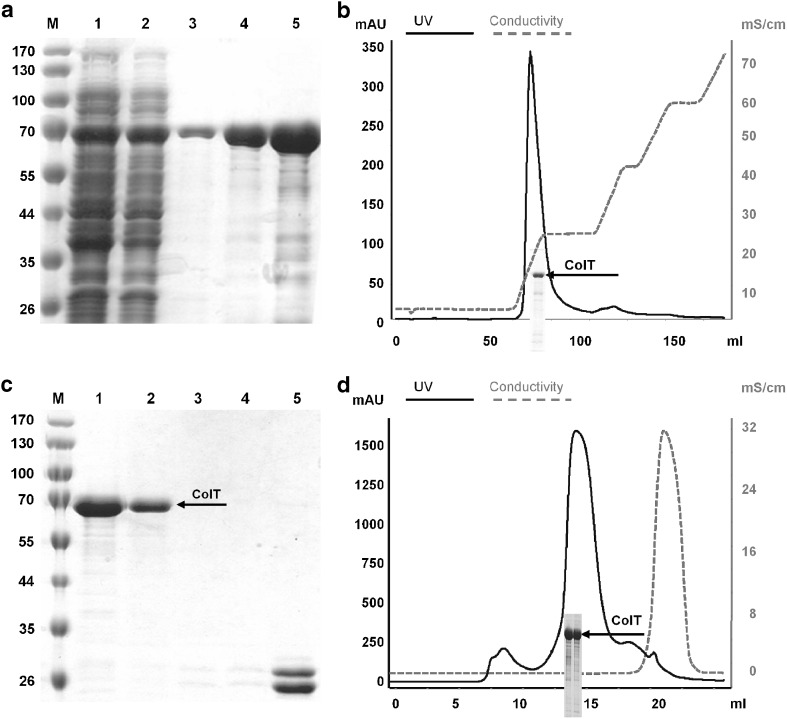
Purification of the catalytic domain of ColT (hT1). For the nomenclature, compare Table 1. *M* prestained protein ladder. **a** Native Ni-NTA purification of hT1 analyzed by SDS-PAGE: *Lane 1* column flow-through; *lanes 2*, *3* wash 1, 2; *lanes 4*, *5* elutions containing hT1. **b** Ion exchange chromatography of hT1. This purification step occurred via ÄKTA FPLC system using a Q-Sepharose column. The protein was eluted with ~100 mM CaCl_2_. **c** Removal of the His_6_-tag from hT1. *Lanes 1*, *2* flow-through containing the target protein without His_6_-tag; *lanes 3*, *4* wash steps; *lane 5* elutions containing the TEV-protease; **d** Size exclusion chromatography of the catalytic domain without His_6_-tag. The first UV peak corresponds to the void volume of the column (Superdex 200 10/300 GL) and the second to the catalytic domain of ColT

To summarize the individual purification steps: (1) because all constructs featured an N- or C-terminal His_6_-tag, immobilized metal affinity chromatography (IMAC) was by default used as initial purification step (Fig. 6a). Most *E. coli* proteins can be easily removed by this method, and as a welcome side effect the target protein is concentrated. (2) Constructs equipped with an additional affinity tag (MBP) were subsequently purified via amylose resin allowing for efficient pooling of undegraded protein with clearly defined termini. (3) To achieve high purity grade protein samples, it was necessary (with few exceptions) to employ another orthogonal purification method. For this purpose we established a calcium gradient ion exchange chromatography, which capitalizes on the specific calcium binding properties of these enzymes. Target proteins were eluted with approximately 100–125 mM CaCl_2_ and analyzed by SDS-PAGE (Fig. 6b). (4) Prior or after IEC the N-terminal tags were removed and samples were re-purified by IMAC. In case of ColH and T, it was necessary to deviate from standard protocols and to increase incubation time (up to 48 h) and the molar ratio (up to 5:1) for complete tag removal without observing any undesired “star” activity (Fig. 6c). (5) Size exclusion chromatography was performed as a final purification step. A maximum of 10 mg protein per run was loaded. All proteins migrated as monomers at the expected molecular size. A representative SEC run is shown in Fig. 6d. (6) Based on the established protein production and purification platform, we succeeded in obtaining monodisperse and mono-isoformic protein samples for all variants of ColG, H, and T.

### Enzymatic activity

After the initial IMAC purification step enzymatic activity of the heterologously expressed enzymes was routinely checked and confirmed by the FALGPA assay (Van Wart and Steinbrink 1981). All variants except the two mini-catalytic domains were active against the synthetic substrate. Consistently, ColH constructs showed the highest overall activity, followed by ColT and ColG. Representative turnover numbers after SEC are given for the catalytic domains of ColH, ColT, and ColG: *k*_cat_/*K*_M_ = 59,100, 7,470, and 130 s^−1^ M^−1^, respectively (Eckhard et al. 2009). As expected, enzyme activity of all variants could be reversibly inhibited by the zinc-specific inhibitor 1,10-phenanthroline (data not shown).

## Discussion

The continuing progress in biotechnological and medical research is uncovering an ever-growing number of possible applications for clostridial collagenases, in particular ColG and H (Antonioli et al. 2007; Chu 1987; Jin et al. 2005; Jordan 2008; Ku et al. 1993; Kuriyama et al. 2001; Segev et al. 2005; Wang et al. 2004). There are various commercial preparations available, but these usually contain several collagenase isoforms and even other proteases (e.g., clostripain). Their heterogeneity and the difficulty of obtaining mono-isoformic preparations have so far hindered us from exhausting their full biotechnological and medical potential, such as the development of application-specific mutants and rational drug design. Therefore, we wanted to develop a protein production and modular purification platform that (1) results in large quantities of highly pure mono-isoformic collagenase samples, (2) is easy to handle, and (3) straightforward to establish, in short a “kit-like technology.” By means of the naturally occurring isoforms accomplished by a molecular dissection of ColG, H, and T, we could show here the feasibility of our efforts (Table 1).

We employed the working horse of protein expression—*E. coli*—and could, thus, fully profit from its properties, such as its well-understood genetics, inexpensive media, fast growth rate, and high expression yields (Jana and Deb 2005; Makrides 1996). To optimize the yield of soluble protein, we tested several expression parameters for all constructs: (1) *E. coli* strains with slightly altered expression characteristics, (2) point of induction, (3) final IPTG concentration, (4) cultivation temperature, and (5) induction period. All monitored parameters affected the soluble protein yield, justifying this comprehensive screening of heterologous expression as the first point of optimization in protein production and purification.

In a second step, we optimized the purification protocols based on a flexible arrangement of different chromatographic techniques (affinity, ion exchange and size exclusion chromatography) to the individual needs of the investigated isoforms (Fig. 4). For instance, a two step purification approach was already sufficient for shorter constructs, e.g., the catalytic domain of ColG (Eckhard et al. 2008), whereas a four-step approach was necessary for the full-length collagenases to meet our purity requirements.

Already at an early stage of this project, we observed the degradation of several constructs by SDS-PAGE analysis and Western blot (data not shown). The revealed (auto)degradation pattern of the full-length constructs apparently correlated roughly with the size of the naturally occurring isoforms and, therefore, gave additional impetus to the idea to mimic their mosaic domain architecture with our constructs. To cope with the degradation problem, we included a tandem affinity tagging strategy for the longer constructs (Table 1 and Fig. 4) by fusing a cleavable maltose binding protein-tag to the N-terminus and a non-cleavable His_6_-tag to the C-terminus. This approach enabled us to pool nondegraded protein in the successive purification steps. In response to the presence of a truncated fragment smaller than the catalytic domain construct in preparations of ColT, two shorter variants were cloned to identify an even smaller active catalytic domain. However, both mini-constructs showed no catalytic activity in the FALGPA assay, although they migrated on SEC at the expected size, indicative of proper folding (data not shown). Therefore, we consider the present S1 variants as minimal versions of the catalytic domains. These observations, (1) C-terminally truncated isoforms in vivo (Bond and Van Wart 1984a; Matsushita et al. 1999; Yoshihara et al. 1994), (2) time dependent degradation of recombinant full-length constructs, and (3) inactivity of C-terminally truncated, “mini” catalytic domains, indicate that (auto-)degradation plays, similar as observed for trypsin (Halangk et al. 2002), an important role in the regulation of clostridial collagenases.

In conclusion, we succeeded in establishing a generic expression and purification strategy for clostridial collagenases in *E. coli* and implemented this strategy for three important and representative examples. This system evades the problems associated with homologous expression (i.e., co-purification of other clostridial toxins) and utilizes the most widely used of all prokaryotic organisms for recombinant protein expression in pharmaceutical industry with well-established GMP standards (Fukui et al. 1989; Yamamoto et al. 2002). Based on the old but by far not outdated workhorse *E. coli*, we can readily provide milligram-amounts of high-grade (contamination-free, monodisperse, mono-isoformic, and conformationally homogeneous) protein preparations needed in diverse biotechnological fields and for various sophisticated downstream applications. Moreover, based on this platform, a systematic screening of chimeric constructs and of other mutants is feasible which will help to identify key residues and motifs in collagenolysis and enlighten the biochemical function of the individual domains present in clostridial collagenases.
